# 2382. Sociodemographic and Occupational Characteristics Associated with Delayed and Low COVID-19 Vaccine Uptake Among Healthcare Personnel: Monroe County, NY

**DOI:** 10.1093/ofid/ofad500.2002

**Published:** 2023-11-27

**Authors:** Savanah Russ, Christopher J Myers, Erin Licherdell, Acacia Bowden, Ellen Chinchili, Runda Dahhan, Christine Hurley, Edwin VanWijngaarden, Ghinwa Dumyati

**Affiliations:** Center for Community Health & Prevention, University of Rochester Medical Center, Rochester, New York; University of Rochester, Rochester, New York; University of Rochester Medical Center, Rochester, New York; University of Rochester School of Medicine and Dentistry, Rochester, New York; University of Rochester School of Medicine & Dentistry, Rochester, New York; University of Rochester, Rochester, New York; University of Rochester, Center for Community Health and Prevention, Rochester, New York; University of Rochester School of Medicine & Dentistry, Rochester, New York; New York Emerging Infections Program and University of Rochester Medical Center, Rochester, New York

## Abstract

**Background:**

Healthcare personnel (HCP) have remained at high risk for SARS-CoV-2 infection during the COVID-19 pandemic. High and timely COVID-19 vaccine uptake is crucial to protect this at-risk population. Our study aims to characterize HCP who delayed initiation of the primary series, and those who did not receive a booster dose.

**Methods:**

Data for this analysis came from a cohort of HCP working at a large healthcare system in Monroe County, NY identified between 12/28/2020-12/01/2022. HCP were enrolled as part of the CDC Emerging Infections Program COVID-19 vaccine effectiveness study using a test-negative case-control design. Participants completed a standardized questionnaire assessing demographic and occupational characteristics. Verified COVID-19 vaccination history for each HCP was collected during the study period. HCP were categorized as having early or delayed vaccine initiation if they received their 1^st^ mRNA COVID-19 vaccine between 12/14/2020-03/30/2021 (early) or 04/01/2021-09/28/2021 (delayed) after which employee vaccine mandates were implemented at this hospital system. HCP were also categorized as having received a 3^rd^ mRNA COVID-19 booster dose or not after 09/24/2021. Logistic regression models were run to identify characteristics of HCP who delayed 1^st^ dose receipt or did not receive a booster dose.

**Results:**

Across the study period, 3,471 HCP were enrolled. Of these, 86.0% had early initiation of their 1^st^ mRNA COVID-19 vaccine, and 82.8% received an mRNA booster dose. Low education, low household income, younger age (< 50), non-White race and public health insurance were all significant predictors of delayed receipt of 1^st^ dose and lack of uptake of a booster. However, advanced professional role was only found to be a significant predictor of early 1^st^ dose receipt (Figure 1). Sensitivity analyses, run by changing dates of early vs delayed initiation to later time points, validated these results.

**Figure 1.**
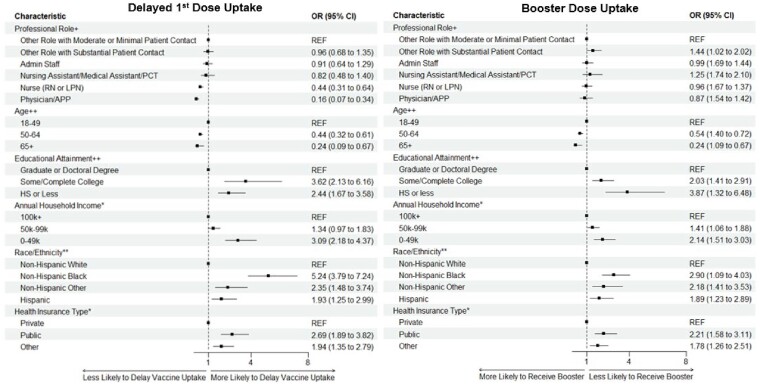
Sociodemographic and Occupational Characteristics Associated with COVID-19 Vaccine Coverage Among Healthcare Personnel (N=3,471)

+Adjusted for gender, race/ethnicity, and education. ++Adjusted for gender and race/ethnicity. *Adjusted for professional role, educational attainment, and race/ethnicity. **Adjusted for gender, age, educational attainment, professional role, annual household income, and health insurance type. Other Role with Moderate or Minimal Patient Contact includes: Nonphysician behavioral health provider, chaplain, care coordinator, dietician, environmental services personnel, food services personnel, patient transport personnel, research personnel, social worker, student, facilitates maintenance personnel, medical equipment technician, laboratory personnel or pharmacist. Other Role with Substantial Patient Contact includes: Dental healthcare provider, emergency medical services personnel, occupational therapist, physical therapist or assistant, phlebotomist, respiratory therapist, radiology technician, speech-language pathologist, and surgical, medical or emergency technician.

**Conclusion:**

Sociodemographic characteristics, rather than occupational, were predictive of high and timely vaccine uptake. Continual monitoring of COVID-19 vaccine uptake among HCP to identify those less likely to receive new booster doses will be crucial for maintaining high vaccination rates in this important population.

**Disclosures:**

**Ghinwa Dumyati, MD**, Pfizer: Grant/Research Support

